# AMGAA: Attention-Guided Multi-Target Generative Adversarial Attack for Vision Transformers

**DOI:** 10.3390/e28060680

**Published:** 2026-06-12

**Authors:** Dongbo Ou, Jintian Lu, Shihui Zhou, Ying Zeng, Dongwan Liao, Haoyin Liu, Chao Yang, Yingsheng He, Jie Tian

**Affiliations:** School of Computer Science and Engineering, Jishou University, Jishou 416000, China; oudongbo@stu.jsu.edu.cn (D.O.); lujd6@mail2.sysu.edu.cn (J.L.); zhoushihui@stu.jsu.edu.cn (S.Z.); zengying@stu.jsu.edu.cn (Y.Z.); 2023400638@stu.jsu.edu.cn (D.L.); liuhaoyin@stu.jsu.edu.cn (H.L.); chaoy@jsu.edu.cn (C.Y.); hysuser@163.com (Y.H.)

**Keywords:** vision transformer, adversarial attack, multi-target attack, transfer attack, attention mechanism, security of AI

## Abstract

Vision Transformers (ViTs) have achieved strong performance in computer vision, but their adversarial robustness remains underexplored. Existing ViT-oriented attacks mainly rely on iterative optimization, leading to high generation cost and limited transferability. Moreover, most generative attacks target a single class, making them inefficient for multi-target scenarios. To address these issues, we propose Attention-Guided Multi-Target Generative Adversarial Attack (AMGAA). AMGAA leverages ViT self-attention to guide target feature fusion and adaptively selects important source patches for perturbation generation. It jointly optimizes adversarial, attention constraint, and total variation losses to improve targeted attack success while preserving visual naturalness. Experiments on CIFAR-10 and ImageNet show that AMGAA achieves average attack success rates (ASRs) of 43.2% and 39.0% in ImageNet single-target transfer attacks and CIFAR-10 multi-target attacks, respectively. Compared with the generative attack methods evaluated in our experiments, AMGAA improves ASR by 5.7 percentage points in the multi-target setting and by 8.1 percentage points in unknown-class generalization. AMGAA also obtains a low LPIPS of 0.018, indicating good visual imperceptibility. Ablation studies confirm the effectiveness of its key components.

## 1. Introduction

With the rapid development of artificial intelligence, deep learning models have shown strong potential in various fields, such as image recognition [1,2,3], automated control [4,5,6], and medical diagnosis [7,8,9]. However, recent studies have revealed that these models are highly vulnerable to adversarial examples, making adversarial robustness a central issue in AI security [10,11]. Adversarial examples are crafted by adding small perturbations to clean inputs. Although such perturbations are often imperceptible to humans, they can mislead deep models into making incorrect predictions. This phenomenon exposes the robustness limitations of deep neural networks and motivates further research on secure model design. Therefore, studying adversarial example generation is essential for understanding model vulnerabilities and developing more robust learning systems.

As a mainstream visual modeling paradigm, Vision Transformer (ViT) has achieved remarkable progress in computer vision [12]. Unlike conventional convolutional neural networks (CNNs), which mainly rely on local convolutions [13,14,15], ViT captures long-range dependencies through self-attention and thus learns more discriminative visual representations. It has achieved state-of-the-art performance on various vision tasks. Therefore, investigating the adversarial vulnerability of ViTs is crucial for their reliable deployment in real-world applications.

Existing studies have shown that, due to the structural properties of ViT self-attention, several attacks effective on CNNs suffer a clear performance drop when transferred to ViTs [16,17]. Inspired by word-substitution attacks against Transformers in natural language processing (NLP), patch-level attack paradigms have been introduced for ViTs [18]. These methods can significantly disrupt ViT predictions by modifying only a small number of image patches, and such patch-wise perturbations are often more effective against ViTs than CNNs. According to the generation mechanism, existing methods can be roughly divided into two categories. The first category iteratively optimizes selected patches using gradients from the target model. These methods usually achieve high white-box attack success rates, but suffer from high computational cost, overfitting, and limited transferability [18,19,20]. The second category relies on generative models, where a trained generator directly outputs adversarial patches in a single forward pass. Such methods are more efficient at inference time and often exhibit better transferability [21]. However, generative attacks for ViTs remain underexplored, and most existing methods are designed for a single target class. A new target class therefore requires retraining, which greatly limits their scalability.

To address these limitations, we propose a multi-target generative adversarial attack framework that fully exploits ViT self-attention to generate adversarial examples with high attack effectiveness and low perceptibility. Specifically, we first extract feature representations and attention weights from both the source and target images. Then, an attention-guided feature fusion module injects discriminative target features into the source representation, encouraging the generated examples to approach the target-class feature distribution. Based on this fused representation, an adaptive perturbation strategy is designed to modulate perturbation generation according to the source attention weights and focus the perturbation on highly attended patches. Finally, we optimize the generation process with attention-aware objectives, including adversarial and attention regularization losses, to balance attack strength and visual imperceptibility. The main contributions of this work are summarized as follows:

1. We propose a novel generative attack framework that extends existing single-target generative attacks to the multi-target setting by exploiting class-shared attention patterns. This design reduces training cost and improves applicability in complex attack scenarios.
2. We incorporate ViT self-attention into target feature fusion and adaptive perturbation generation. Through attention-guided semantic injection and important patch selection, the perturbation is concentrated on model-sensitive regions, improving both attack success rate and transferability while maintaining visual imperceptibility.
3. We conduct extensive experiments on ImageNet and CIFAR-10 to evaluate AMGAA under single-target transfer, multi-target attack, and unknown-class generalization settings. The results show that AMGAA achieves higher average ASR across multiple Transformer and CNN victim models, while preserving strong perceptual quality in terms of SSIM and LPIPS.

## 2. Related Work

### 2.1. Development of Adversarial Example Generation

The concept of adversarial examples was first introduced by Szegedy et al. [10], who showed that deep neural networks (DNNs) are vulnerable to imperceptible input perturbations. This finding exposed the inherent weakness of neural networks and motivated extensive research on adversarial attacks. Goodfellow et al. [11] proposed the Fast Gradient Sign Method (FGSM), which efficiently generates adversarial examples using the gradient of the loss with respect to the input. Carlini and Wagner later introduced the C&W attack [22], which improves imperceptibility by explicitly optimizing the distance between adversarial and clean samples. Madry et al. [23] proposed Projected Gradient Descent (PGD), a strong iterative attack that crafts adversarial examples through multi-step optimization. Dong et al. [24] further developed Momentum Iterative FGSM (MI-FGSM), which incorporates momentum to stabilize gradient updates and enhance transferability. To provide a more reliable robustness evaluation, Croce and Hein proposed AutoAttack [25], an automated ensemble of parameter-free attacks. Although these methods have achieved strong performance on CNNs, their applicability and effectiveness against attention-based Vision Transformers require further investigation.

### 2.2. Vision Transformers and Adversarial Attacks

With the wide adoption of Vision Transformers in visual recognition, their robustness against adversarial attacks has attracted increasing attention. Naseer et al. found that ViTs can be more robust than CNNs in certain adversarial settings, possibly due to the global feature modeling capability of self-attention [26]. However, whether ViTs are consistently more robust than CNNs remains controversial.

Inspired by token-level attacks against Transformers in NLP, Fu et al. proposed Patch-Fool for ViTs [18]. This method disrupts self-attention by applying tailored perturbations to critical image patches. The results show that ViTs may be more vulnerable than CNNs under patch-level attacks, indicating that their robustness advantage does not hold in all attack scenarios. Following this line, subsequent studies [19,27] further analyzed ViT robustness from the perspective of patch/token perturbations. Overall, patch- and token-level attacks have become an important direction for studying the adversarial vulnerability of ViTs and for understanding their self-attention and token representation properties.

### 2.3. Attention Mechanisms in Adversarial Example Generation

Attention mechanisms were first widely used in sequence modeling tasks, such as machine translation, due to their ability to capture long-range dependencies. With the emergence of self-attention-based vision models such as ViT, attention has also been systematically introduced into computer vision. Compared with conventional convolutional features, self-attention explicitly models correlations among spatial locations and semantic regions, providing more fine-grained and structured priors for adversarial example generation.

For example, Wu et al. [28] incorporated internal attention maps into the attack objective and constrained perturbations to high-attention regions, significantly improving the transferability of adversarial examples across models. Wang et al. [29] used attention maps to select a small set of salient pixels as optimization variables and searched for perturbations in this reduced subspace with a large-scale multi-objective evolutionary algorithm, achieving a better balance between black-box attack success rate and visual quality. Sharma et al. [30] exploited intermediate attention maps of VQA models to apply targeted perturbations to image-text alignment regions, enabling adversarial examples to alter model answers while preserving visual naturalness.

These studies demonstrate that attention plays an important role in generating high-quality adversarial examples. For models inherently equipped with self-attention, such as ViTs, effectively exploiting this mechanism is crucial for improving attack effectiveness and perceptual quality.

### 2.4. Adaptive Perturbation Generation

Adaptive perturbation generation is an effective strategy for improving adversarial attack performance. Existing studies have explored adaptive attacks from different perspectives, including perturbation magnitude, perturbation region, and optimization step size. To address the image-dependent variation in optimal scaling factors, Yuan et al. [31] designed an adaptive perturbation strategy that selects a suitable perturbation strength for each image, improving attack success rate while reducing computational cost. To overcome the limited scale adaptability of local adversarial perturbations, Duan et al. [32] proposed a region-adaptive local perturbation method that adjusts the perturbation region according to object size and shape, thereby improving attack generality.

Furthermore, Liu et al. [33] combined local mixup with adaptive step size to enhance input diversity and dynamically adjust perturbation updates, improving cross-model transferability. From the optimization perspective, Hu et al. [34] proposed APDL, which adaptively adjusts the single-step perturbation magnitude and adopts dual-loss optimization to accelerate convergence while maintaining a high attack success rate with fewer iterations. However, these methods are mainly designed for CNNs or specific vision tasks and do not fully consider the patch-token representation and self-attention mechanism of ViTs. Therefore, adaptive perturbation generation for ViTs remains underexplored.

### 2.5. Multi-Target Generators

Generative adversarial attacks use a single feed-forward generator to directly produce perturbations or adversarial examples, avoiding the cost and overfitting risk of iterative optimization and improving efficiency in black-box settings. Representative methods include AdvGAN [35] and TTP [36]. AdvGAN employs a generative adversarial network (GAN) to approximate the data distribution and generate high-quality adversarial examples, while TTP improves transferability by matching the distribution of perturbed images to that of the target class, reducing reliance on model-specific decision boundaries.

However, these methods are usually designed for a single target class, requiring a separate generator for each new class and thus increasing training cost. To address this issue, MAN [37] introduced a multi-target generator, where the discrete target label t∈{1,…,K} is explicitly used as a condition. It extracts image appearance features and target-label features separately, fuses them in the feature space, and uses residual and deconvolutional layers to generate adversarial examples for the given target label. As a result, one generator can cover *K* target classes while maintaining attack success rate and transferability. Building on this idea, CGNC [38] incorporates CLIP-based textual semantics into the multi-target generator and uses cross-attention to enhance the interaction between target semantics and image features, improving transferable targeted attacks.

Overall, existing generative methods have demonstrated the feasibility of “one generator for multiple targets” mainly in CNN-based settings. For ViTs, how to exploit class-shared attention patterns to build an efficient and transferable multi-target generator remains insufficiently explored.

## 3. Methodology

### 3.1. Overview of the Framework

**Figure 1:**
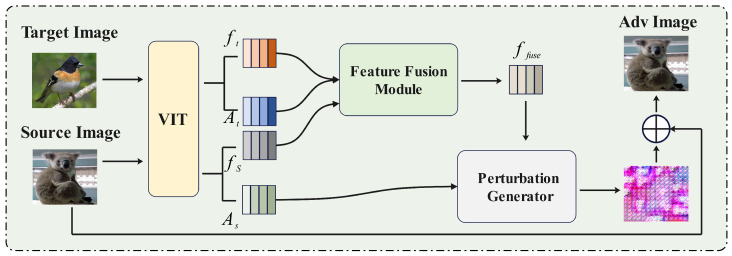
Overall framework of AMGAA. The source and target images are first fed into ViT to extract feature representations and attention maps. The target features and attention information are used by the feature fusion module to generate target-guided fused features, while the source attention map guides the perturbation generator to produce adaptive perturbations on important regions. The generated perturbation is added to the source image to obtain the final adversarial example.

Our framework is built on Vision Transformer (ViT) and integrates feature fusion with perturbation generation to form an efficient adversarial example generation pipeline. As shown in Figure 1, the framework consists of four main modules:

- ViT feature extraction module: This module extracts deep feature representations and attention weights from the source and target images, providing the basis for feature alignment and perturbation generation.
- Feature fusion module: Discriminative target features are selected using target attention information and injected into the source representation to enhance feature-level similarity to the target class.
- Perturbation generation module: Guided by the source attention distribution, this module adaptively concentrates perturbations on important regions, improving attack precision and effectiveness.
- Loss optimization module: This module optimizes the model from multiple perspectives. Specifically, adversarial loss, attention constraint loss, and total variation loss are jointly optimized to improve attack performance, structural consistency, and visual naturalness.

By coordinating these modules, the proposed method effectively exploits the global modeling capability of ViT, enabling the generated adversarial examples to better approach the target feature distribution while maintaining high visual imperceptibility.

### 3.2. ViT Feature Extraction

As a core component of the framework, the feature extraction module captures deep feature representations and global attention weights from input images. Built on Vision Transformer (ViT), this module first divides an input image into fixed-size patches and then models the patch sequence with Transformer encoders to obtain discriminative feature and attention representations.

Given a source image $x_{s}$ and a target image $x_{t}$, the ViT-based feature extraction process is formulated as:

(1) $$F_{s},A_{s}=\mathrm{ViT}\left(x_{s}\right),$$

(2) $$F_{t},A_{t}=\mathrm{ViT}\left(x_{t}\right),$$

where $F_{s},F_{t}\in R^{n\times d}$ denote the feature representations of the source and target images, respectively. Here, *n* is the number of patches and *d* is the feature dimension. $A_{s},A_{t}\in R^{n\times n}$ denote the corresponding self-attention matrices, which describe patch-wise correlations and attention distributions.

Different ViT layers capture hierarchical information. Shallow layers mainly encode local textures and fine-grained details, whereas deeper layers tend to capture high-level semantic information. To exploit multi-level representations, AMGAA does not rely on a single Transformer layer. Instead, it introduces a weighted aggregation mechanism to integrate features and attention maps from multiple layers:

(3) $$F_{\mathrm{final}}=\sum_{l=1}^{L}w_{l}\cdot F^{\left(l\right)},$$

(4) $$A_{\mathrm{final}}=\sum_{l=1}^{L}w_{l}\cdot A^{\left(l\right)},$$

where *L* denotes the number of selected Transformer layers, and $w_{l}$ is the learnable weight of the *l*-th layer, satisfying

(5) $$\sum_{l=1}^{L}w_{l}=1.$$

In these equations, $F^{\left(l\right)}$ and $A^{\left(l\right)}$ represent the feature representation and attention matrix from the *l*-th layer, respectively. This multi-layer aggregation preserves both local details and global semantics, providing a more robust representation for subsequent attention-guided feature fusion and perturbation generation.

### 3.3. Attention-Guided Feature Fusion

To move the generated adversarial examples closer to the target image in the feature space, we design an attention-guided feature fusion mechanism. This mechanism weights the target features using the target attention distribution and injects key semantic information into the source representation, thereby enhancing the tendency of the source image to shift toward the target class.

Specifically, for the *i*-th patch of the source image, the fused feature is defined as:

(6) $$F_{s}^{\prime }\left(i\right)=F_{s}\left(i\right)+\alpha {\hat{A}}_{t}\left(i\right)F_{t}\left(i\right),$$

where $F_{s}\left(i\right)$ and $F_{t}\left(i\right)$ denote the feature vectors of the *i*-th patch in the source and target images, respectively. ${\hat{A}}_{t}\left(i\right)$ is the normalized importance weight of the *i*-th patch derived from the target attention matrix $A_{t}$, and α is a hyperparameter controlling the feature injection strength.

In this fusion process, target regions with higher attention weights exert stronger influence on the source representation, encouraging the source features to approach the discriminative features of the target image. Unlike direct global feature replacement, this attention-guided selective injection highlights key target regions more precisely, improves feature transfer effectiveness, and provides a clearer direction for subsequent perturbation generation.

### 3.4. Adaptive Perturbation Generation Module

After feature fusion, we further design an attention-based adaptive perturbation generation strategy. Unlike conventional methods that apply perturbations uniformly over the entire image, our method determines the perturbation regions according to the source attention response. This allows the limited perturbation budget to focus on decision-sensitive patches, improving attack efficiency and effectiveness.

First, the source attention matrix $A_{s}$ is aggregated to compute the global importance score of each patch:

(7) $$s_{i}=\sum_{j}A_{s}\left(j,i\right),$$

where $s_{i}$ denotes the importance of the *i*-th patch in global attention interactions. Based on $s_{i}$, the top-*k* important patches are selected, and their index set is denoted as $\Omega_{k}$. A binary mask is then constructed as:

(8) $$M\left(i\right)=\left\{\begin{array}{cc} 1, & i\in \Omega_{k}\\ 0, & i\notin \Omega_{k} \end{array}\right..$$

Then, the feature variation before and after fusion is used to construct the patch-level guidance representation:

(9) $$z\left(i\right)=M\left(i\right)\cdot {\hat{A}}_{s}\left(i\right)\cdot \left(F_{s}^{\prime }\left(i\right)-\beta F_{s}\left(i\right)\right),$$

where ${\hat{A}}_{s}\left(i\right)$ denotes the normalized importance weight of the *i*-th source patch, and $F_{s}^{\prime }\left(i\right)-\beta F_{s}\left(i\right)$ represents the feature variation with source-feature retention controlled by the learnable coefficient β. The guidance representation z(i) is not directly added to the image. Instead, it serves as the input condition of the perturbation generator.

**Figure 2:**
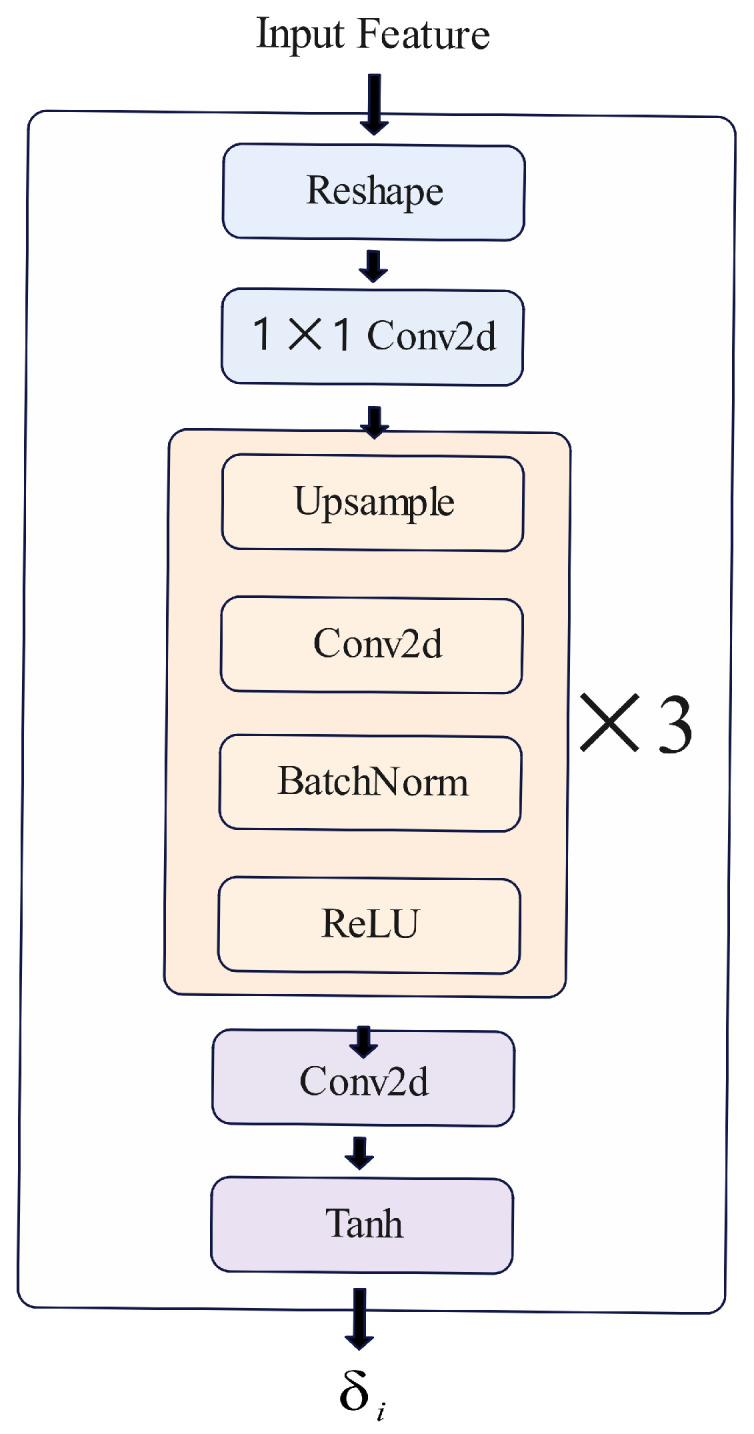
Architecture of the perturbation generator.

Given the perturbation budget ϵ, the pixel-level perturbation is generated by:

(10) $$\delta =\mathrm{clip}\left(G_{\theta }\left(z\right),-\epsilon ,\epsilon \right),$$

where $G_{\theta }\left(\cdot \right)$ (see Figure 2) denotes the perturbation generator, which maps the patch-level guidance representation to a pixel-level perturbation with the same spatial size as the source image. The function clip(·) constrains the generated perturbation within the budget [−ϵ,ϵ].

Finally, the adversarial example is obtained as:

(11) $$x_{s}^{\prime }={\mathrm{clip}}_{\left[0,1\right]}\left(x_{s}+\delta \right).$$

This design ensures that the perturbation is generated in the pixel space while still being guided by feature variation, attention strength, and the selected patch mask. As a result, the perturbation is concentrated on highly important regions, making it more consistent with the attention characteristics of ViTs and improving both attack effectiveness and visual imperceptibility.

### 3.5. Loss Optimization

To balance attack effectiveness, attention consistency, and visual naturalness, we construct a joint optimization objective consisting of adversarial loss, attention constraint loss, and total variation loss. By combining these losses, the proposed method improves targeted attack success while suppressing unnecessary high-frequency noise.

#### 3.5.1. Adversarial Loss

The adversarial loss encourages the generated adversarial example to approach the target image in the feature space. We adopt cosine similarity loss to measure the feature distance between the adversarial example and the target image:

(12) $$L_{\mathrm{adv}}=1-\frac{F\left(x_{s}^{\prime }\right)\cdot F\left(x_{t}\right)}{∥F\left(x_{s}^{\prime }\right)∥\;∥F\left(x_{t}\right)∥},$$

where $F(x_{s}^{\prime })$ and $F(x_{t})$ denote the feature representations of the adversarial example and the target image, respectively. Minimizing this loss drives the adversarial example toward the target feature distribution, thereby improving targeted attack effectiveness.

#### 3.5.2. Attention Constraint Loss

To align the attention distribution of the adversarial example with that of the target image, we introduce an attention constraint loss:

(13) $$L_{\mathrm{attn}}=\sum_{i=1}^{n}{∥{\hat{A}}_{x_{s}^{\prime }}\left(i\right)-{\hat{A}}_{t}\left(i\right)∥}^{2},$$

where ${\hat{A}}_{x_{s}^{\prime }}\left(i\right)$ denotes the normalized attention weight of the adversarial example at the *i*-th patch, and ${\hat{A}}_{t}\left(i\right)$ denotes the corresponding normalized attention weight of the target image. Minimizing this loss encourages attention-level alignment and enhances target-oriented guidance.

#### 3.5.3. Total Variation Loss

To reduce high-frequency noise and improve visual naturalness, we employ total variation (TV) loss as a smoothness regularizer:

(14) $$L_{\mathrm{tv}}=\sum_{i,j}\sqrt{{\left(x_{i+1,j}^{\prime }-x_{i,j}^{\prime }\right)}^{2}+{\left(x_{i,j+1}^{\prime }-x_{i,j}^{\prime }\right)}^{2}},$$

where $x_{i,j}^{\prime }$ denotes the pixel value of the adversarial example at position (i,j). TV loss penalizes abrupt changes between adjacent pixels, encouraging spatially smoother perturbations and reducing visually noticeable artifacts.

#### 3.5.4. Overall Objective

The overall loss function is defined as:

(15) $$L_{\mathrm{total}}=L_{\mathrm{adv}}+\lambda_{1}L_{\mathrm{attn}}+\lambda_{2}L_{\mathrm{tv}},$$

where $\lambda_{1}$ and $\lambda_{2}$ are weighting coefficients that balance the contributions of different loss terms.

During training, the generator parameters are updated by backpropagation, rather than iteratively optimizing the perturbation for each image. Under the perturbation budget constraint, the generator learns to produce adversarial perturbations that balance attack effectiveness, attention transfer, and visual imperceptibility. After training, adversarial examples can be generated with a single forward pass, improving attack efficiency.

## 4. Experiments and Results

### 4.1. Experimental Settings

To comprehensively evaluate the effectiveness, transferability, and generalization ability of AMGAA, we conduct extensive experiments and compare it with several representative adversarial attack methods.

- Datasets: We use CIFAR-10 [39] and ImageNet [40] in our experiments. CIFAR-10 is mainly used for training and evaluating multi-target attacks, while ImageNet is used to evaluate transferability and generalization ability.
- Victim models: To evaluate the attack performance of AMGAA across different architectures, we consider several representative victim models, including the standard ViT-B/16 [12], Swin Transformer (Swin-B) [41] with hierarchical window-based attention, robustness-oriented RVT [42], and classical CNNs, including ResNet-50 [15], DenseNet-121 [43], and VGG-19 [14]. These models cover both Transformer and CNN-based architectures, enabling a systematic evaluation of cross-architecture transferability and attack effectiveness.
- Evaluation metrics: We evaluate the proposed method using three metrics.
  1. Attack Success Rate (ASR): the proportion of adversarial examples that successfully induce the target model to make the desired incorrect prediction.
  2. Generalization ability: the ability of the generator to produce effective adversarial examples for unknown target classes.
  3. Perceptual quality: SSIM and LPIPS are used to measure structural similarity and perceptual distance between clean and adversarial images.
- Compared methods: We compare AMGAA with six representative adversarial attacks, including gradient-based iterative pixel-level attacks PGD [23], C&W [22], and MI-FGSM [24], the local patch-based attack G-Patch [21], and generative multi-target attacks MAN [37] and CGNC [38]. These baselines cover different attack paradigms, allowing a comprehensive comparison in terms of attack success, transferability, and generalization.
- Implementation details: In all experiments, the perturbation budget is set to 16/255, and ViT-B/16 is used as the surrogate model. For AMGAA, we use the AdamW optimizer [44] with a learning rate of 2 × 10^−4^ and train the model for 20 epochs. The number of selected patches *k* is set to 16, and the feature injection coefficient α is set to 0.3, and the learnable source-feature retention coefficient β is initialized to 1. For the baseline attacks, PGD is performed for 20 iterations with a step size of 2/255. MI-FGSM is also run for 20 iterations with a step size of 2/255 and a momentum factor of 1.0. C&W uses the Adam optimizer [45] with a learning rate of 0.01 and a maximum of 1000 iterations. Unless otherwise specified, other hyperparameters follow the original papers or common settings, and all methods are evaluated under the same perturbation budget for fair comparison.

### 4.2. Main Experimental Results

#### 4.2.1. Transferability in Single-Target Attacks

We evaluate the transferability of different methods under the single-target setting on ImageNet. Since evaluating only one target class may introduce bias caused by class-specific difficulty, we randomly select 20 target classes for evaluation. For each selected class, it is fixed as the attack target, and ImageNet training images whose ground-truth labels are different from the target class are used as source images. A separate single-target attack model is trained for each target class, resulting in 20 independently trained attack models.

During testing, each trained model is used only to generate adversarial examples for its corresponding target class. For each target class, we randomly sample 10,000 source images from the ImageNet validation set, ensuring that their ground-truth labels are different from the current target class. Therefore, a total of 200,000 targeted adversarial examples are generated for evaluation. All adversarial examples are first generated using ViT-B/16 as the surrogate model and then transferred to Swin-B, RVT, DenseNet-121, VGG-19, and ResNet-50 for black-box evaluation. For fair comparison, all methods are evaluated under the same perturbation budget. For G-Patch, the patch size is set to 64×64. For AMGAA, we set k=16. Under an input size of 224×224 and a patch size of 16×16, the perturbed area is equivalent to a 64×64 patch. For CGNC and MAN, we follow the hyperparameter settings reported in their original papers. The final results are reported as the average ASR over the 20 target classes.

As shown in Table 1, AMGAA achieves the best overall performance in the single-target transfer attack setting, with an average ASR of 43.2%. It clearly outperforms CGNC (37.1%), G-Patch (35.9%), and MAN (33.9%). On the surrogate model ViT-B/16, AMGAA achieves an ASR of 91.4%, demonstrating its ability to generate strongly target-oriented adversarial examples. More importantly, in black-box transfer evaluation, AMGAA obtains the highest ASR on Swin-B, RVT, DenseNet-121, and VGG-19, reaching 55.4%, 40.7%, 30.1%, and 27.6%, respectively. These results indicate that AMGAA has strong cross-model transferability. Although G-Patch and MAN slightly outperform AMGAA on ResNet-50, AMGAA still shows more stable overall performance across most victim models. These results demonstrate that exploiting ViT attention for target feature fusion and adaptive perturbation allocation can effectively improve the transferability and overall effectiveness of generative targeted attacks.

#### 4.2.2. Comparison of Multi-Target Attack Success Rate

We evaluate the attack success rate of different methods in the multi-target setting on CIFAR-10. Specifically, models are trained on the CIFAR-10 training set and evaluated on the CIFAR-10 test set. During testing, we randomly select 5000 images from the test set. For each image, all nine classes different from its ground-truth label are used as target classes, and the corresponding targeted adversarial examples are generated. This results in 45K adversarial examples for evaluation.

An attack is considered successful if the adversarial example is classified as its assigned target class. Based on this criterion, we compute the targeted attack success rate (ASR). In this experiment, AMGAA is mainly compared with two representative multi-target generative attacks, MAN and CGNC, to evaluate its multi-target attack capability and cross-model transferability.

As shown in Table 2, AMGAA achieves the best overall performance in the multi-target attack setting, with an average ASR of 39.0%, outperforming CGNC (33.3%) and MAN (30.9%). On the surrogate model ViT-B/16, AMGAA reaches an ASR of 89.8%, indicating its strong ability to learn target-oriented perturbations under multiple target classes. For black-box victim models, AMGAA also achieves the best or competitive ASR on Swin-B, RVT, DenseNet-121, VGG-19, and ResNet-50, with scores of 47.4%, 36.8%, 26.4%, 22.1%, and 11.8%, respectively. In particular, AMGAA improves over CGNC by 7.9 and 8.4 percentage points on Swin-B and RVT, respectively, showing stronger transferability across Transformer architectures. Overall, these results demonstrate that AMGAA maintains high targeted attack performance in the multi-target setting and improves transferability across different victim models.

#### 4.2.3. Comparison of Generalization Ability

We evaluate the generalization ability of AMGAA on ImageNet. Specifically, 500 classes are randomly selected as known classes to train MAN, CGNC, and our AMGAA, while the remaining 500 classes are used as unknown target classes. During evaluation, 1000 source images are randomly sampled from the validation set of the known classes. For each source image, one target image is sampled from each unknown class, and the source image is attacked toward the corresponding target class, yielding 500,000 targeted adversarial examples in total. Generalization performance is measured by the attack success rate on these examples.

As shown in Table 3, AMGAA demonstrates stronger generalization ability on unknown classes than the compared methods, achieving an average ASR of 21.1%, which is higher than CGNC (13.0%) and MAN (5.9%). On the surrogate model ViT-B/16, AMGAA reaches an ASR of 41.7%, outperforming CGNC and MAN by 11.2 and 21.0 percentage points, respectively. This indicates that AMGAA can still learn effective target-oriented perturbation patterns for unknown classes. On Swin-B, AMGAA improves over CGNC by 17.6 percentage points, showing stronger transfer generalization in both unknown class and cross-model settings. Overall, these results suggest that AMGAA is not limited to target classes observed during training, but can also maintain effective attack performance on unknown classes, demonstrating better generalization and practical potential.

#### 4.2.4. Perceptual Imperceptibility Analysis

We further evaluate the perceptual imperceptibility of adversarial examples generated by different generative attacks. Specifically, we conduct this analysis using the adversarial examples from the single-target transfer setting in Section 4.2.1. For G-Patch, CGNC, and AMGAA, each method is evaluated on 20 target classes, with 10,000 targeted adversarial examples generated for each class. Thus, 200,000 adversarial examples are used for perceptual quality evaluation for each method.

We adopt SSIM and LPIPS to measure the visual difference between clean and adversarial images. A higher SSIM indicates better structural similarity, while a lower LPIPS indicates stronger perceptual imperceptibility. The final results are obtained by averaging SSIM and LPIPS over the 200,000 adversarial examples generated by each method.

As shown in Table 4, AMGAA achieves the best perceptual imperceptibility. Compared with G-Patch, AMGAA obtains higher SSIM and lower LPIPS. Compared with CGNC, AMGAA achieves a lower LPIPS while maintaining the same SSIM. These results indicate that AMGAA can better suppress visual perturbations and preserve natural image appearance while maintaining strong attack performance.

### 4.3. Ablation Studies

To evaluate the influence of different design factors in AMGAA, we conduct ablation studies from three aspects: module design, loss function, and hyperparameter setting. Unless otherwise specified, all ablation experiments follow the same setting as the multi-target attack experiment in Section 4.2.2, including dataset split, surrogate model, victim models, and perturbation budget, to ensure fair comparison.

#### 4.3.1. Module Ablation Study

To verify the effectiveness of the core modules in AMGAA, we design the following variants:

- Full AMGAA: The complete model.
- w/o attention-guided fusion (w/o AGF): Target attention guidance is removed during feature fusion, while only target feature injection is retained. This setting examines the effect of attention-guided semantic injection.
- w/o adaptive perturbation (w/o AP): Adaptive perturbation weighting is removed, and uniform perturbations are applied to the selected patches. This setting evaluates the role of source-attention-based perturbation allocation.
- Random-k selection (Rand-k): Importance-based patch selection is replaced with random selection of *k* patches. This setting verifies the effectiveness of attention-based patch selection.
- w/o multi-layer aggregation (w/o MLA): Only the last-layer ViT features and attention maps are used. This setting assesses the contribution of multi-layer representation aggregation.

**Figure 3:**
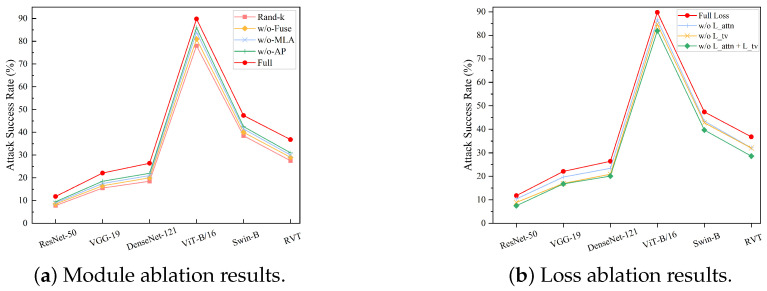
Ablation results of AMGAA on different victim models.

The module ablation results are shown in Figure 3a.

The results show that removing any key component leads to performance degradation, indicating that each module contributes to the final performance of AMGAA. Among all variants, random-*k* selection causes the largest drop in average ASR, from 39.0% to 34.6%, demonstrating the importance of importance-based patch selection for multi-target attacks. Removing attention-guided fusion reduces the average ASR to 35.4%, indicating that target attention guidance effectively enhances semantic injection and improves target-oriented attack performance. In addition, removing adaptive perturbation and multi-layer aggregation decreases the average ASR to 36.8% and 36.1%, respectively, showing that adaptive perturbation allocation and multi-layer feature aggregation are also important for improving attack stability and cross-model transferability.

#### 4.3.2. Loss Ablation Study

The total loss comprises the adversarial loss, the attention constraint loss ($L_{\mathrm{attn}}$), and the total variation loss ($L_{\mathrm{tv}}$). To analyze the effect of each loss term, we design four settings: Full Loss, w/o $L_{\mathrm{attn}}$, w/o $L_{\mathrm{tv}}$, and w/o $L_{\mathrm{attn}}+L_{\mathrm{tv}}$, where both regularization terms are removed and only the adversarial loss is retained. These settings allow us to examine the contribution of each loss term to the attack success rate. The loss ablation results are shown in Figure 3b.

The results show that removing any loss term degrades the attack performance, indicating that all loss components contribute to AMGAA. When $L_{\mathrm{attn}}$ is removed, the average ASR drops from 39.0% to 36.1%, suggesting that the attention constraint loss improves attention consistency between the adversarial and target images and enhances target-oriented transferability. Removing $L_{\mathrm{tv}}$ further reduces the average ASR to 34.9%, showing that total variation loss helps suppress high-frequency noise and stabilize the perturbation structure. When both $L_{\mathrm{attn}}$ and $L_{\mathrm{tv}}$ are removed, the average ASR decreases to 32.9%, indicating that these two regularization terms are complementary in improving attack effectiveness and sample quality.

#### 4.3.3. Hyperparameter Analysis

To further evaluate the influence of key parameter settings, we analyze two core hyperparameters in AMGAA, the number of important patches *k* and the feature injection coefficient α. These two parameters control the spatial coverage of perturbations and the strength of target semantic injection, respectively, and therefore directly affect attack performance and transferability. This analysis helps reveal the performance trends under different parameter settings and provides guidance for selecting the default values.

- Number of important patches *k*: This parameter controls the perturbation coverage. A smaller *k* helps preserve visual naturalness but may limit attack strength, while a larger *k* can improve ASR but may introduce more visible distortions. We evaluate the performance under k∈{1,4,8,16,32,64}.

**Figure 4:**
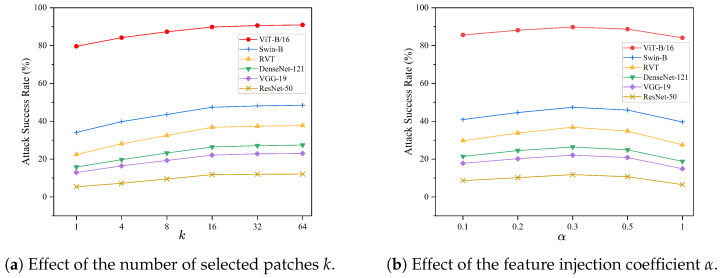
Effects of key hyperparameters on attack success rate across different victim models.

The results in Figure 4a show that the average ASR generally increases as *k* becomes larger, rising from 28.4% at k=1 to 39.9% at k=64. This indicates that increasing the perturbation coverage can enhance attack effectiveness. However, the improvement becomes marginal when *k* increases from 16 to 32 and 64, suggesting that adding more patches does not yield proportional gains. Considering both attack performance and perturbation cost, we set k=16 as the default value.

- Feature injection coefficient α: This parameter controls the strength of target feature injection. A small α may provide insufficient target semantic guidance, while an excessively large α may cause over-aggressive fusion and disrupt feature stability and visual naturalness. We evaluate the effect of α∈{0.1,0.2,0.3,0.5,1.0} on ASR and transferability.

As shown in Figure 4b, α has a significant impact on model performance. As α increases from 0.1 to 0.3, the average ASR improves from 34.0% to 39.0%, indicating that moderate target feature injection enhances semantic guidance. However, when α further increases to 0.5 and 1.0, the performance starts to decline, suggesting that overly strong feature injection may disturb the source feature structure and hinder adversarial generation and transfer. Overall, α=0.3 achieves the best performance and is therefore used as the default setting.

### 4.4. Attention Visualization Analysis

To verify whether the adversarial examples generated by AMGAA can indeed affect the internal attention behavior of ViTs and thereby facilitate the attack, we visualize the attention maps following the method of Kim et al. [46]. Specifically, we average the attention scores across all attention heads in each Transformer layer and visualize the attention score of each token with respect to a given query token, i.e., the center patch marked by the red box in the example. This visualization experiment is conducted based on the generalization setting in Section 4.2.3, and four adversarial examples are selected from the 500,000 generated samples for visualization analysis.

**Figure 5:**
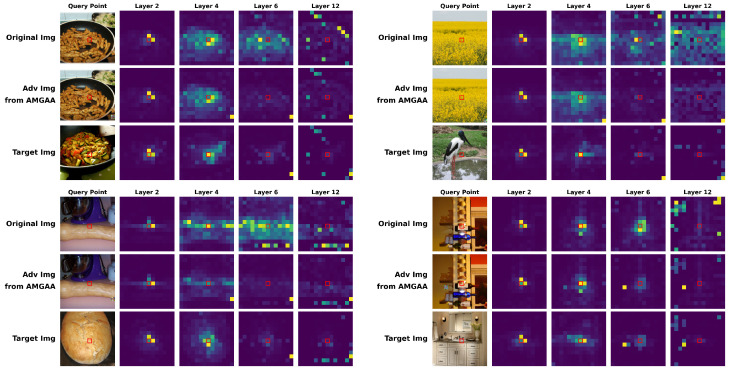
Visualization of ViT attention maps for the original images, AMGAA-generated adversarial images, and target images. The red box denotes the selected query patch. In the attention heatmaps, brighter yellow-green colors indicate stronger attention responses, whereas darker purple-blue colors indicate weaker attention responses. Compared with the original images, the adversarial images preserve similar visual appearance while changing attention responses across different Transformer layers. The target images serve as references for examining whether the adversarial attention responses exhibit target-oriented patterns.

As shown in Figure 5, the adversarial images generated by AMGAA remain visually similar to the original images, but their attention responses differ across different Transformer layers. The target images serve as references for examining whether the adversarial attention responses exhibit target-oriented patterns. In particular, the attention distributions of adversarial images in the middle and deeper layers exhibit noticeable changes compared with the original images and show a tendency to approach the target-related attention patterns. This phenomenon suggests that AMGAA can modify the internal attention behavior of ViTs through visually imperceptible perturbations. These results further support the effectiveness of the proposed attention-guided perturbation generation strategy and provide intuitive evidence that AMGAA facilitates target-oriented adversarial transfer by influencing attention responses.

## 5. Conclusions

In this paper, we propose AMGAA, a multi-target generative adversarial attack method for exploiting the vulnerability of Vision Transformers in targeted attack scenarios. AMGAA leverages ViT self-attention in both feature fusion and perturbation generation, and jointly optimizes adversarial loss, attention constraint loss ($L_{\mathrm{attn}}$), and total variation loss ($L_{\mathrm{tv}}$) to balance attack effectiveness, transferability, and visual imperceptibility. Experimental results show that AMGAA outperforms competing methods in single-target transfer attacks, multi-target attacks, and unknown-class generalization. It also demonstrates clear advantages in cross-model transferability. Perceptual quality evaluation further indicates that AMGAA can maintain strong attack performance while preserving good visual naturalness. Ablation studies confirm the effectiveness of attention-guided feature fusion, adaptive perturbation allocation, important patch selection, and the proposed loss terms. Overall, this work provides an effective framework for multi-target generative attacks against ViTs. Beyond attack generation, AMGAA can also help analyze the adversarial vulnerability of ViTs by revealing how perturbations on decision-sensitive patches influence internal attention responses and target-oriented feature representations. This perspective may provide useful insights for robustness evaluation and the design of more reliable Transformer-based vision models. Nevertheless, AMGAA still has limited generalization capability when attacking unknown target classes, particularly compared with its performance on seen classes. Future work will focus on improving the generalization ability of generative attacks across unknown categories and exploring their applicability in real-world scenarios. In this direction, we will further investigate whether AMGAA-generated adversarial examples can be incorporated into adversarial retraining or fine-tuning of ViT models, with the goal of enhancing robustness against other known attack methods while maintaining standard classification performance on clean images. We will also explore an iterative attack–retraining process, where the retrained model is attacked again to generate new adversarial samples, so as to more systematically evaluate and improve the robustness of ViTs.

## Figures and Tables

**Table 1:** Attack success rates (%) of different methods on various models under the single-target setting. The surrogate model is ViT-B/16.

Attacks	ViT-B/16	Swin-B	RVT	DenseNet-121	VGG-19	ResNet-50	Avg
PGD	96.1	29.6	12.4	11.4	9.9	5.6	27.5
C&W	94.6	20.3	9.1	13.7	8.5	7.4	25.1
MI-FGSM	93.9	14.5	5.3	6.4	4.2	3.8	21.4
G-Patch	70.8	49.7	35.4	24.5	20.1	14.7	35.9
MAN	89.6	40.1	24.3	19.9	15.2	14.4	33.9
CGNC	90.5	47.4	33.2	20.0	19.7	11.6	37.1
AMGAA	91.4	55.4	40.7	30.1	27.6	14.1	43.2

**Table 2:** Multi-target attack success rates (%) on CIFAR-10. The surrogate model is ViT-B/16.

Attacks	ViT-B/16	Swin-B	RVT	DenseNet-121	VGG-19	ResNet-50	Avg
MAN	86.4	36.0	19.7	17.4	14.5	10.9	30.9
CGNC	87.7	39.5	28.4	16.6	18.4	9.4	33.3
AMGAA	89.8	47.4	36.8	26.4	22.1	11.8	39.0

**Table 3:** Generalization performance (%) on unknown classes of ImageNet. The surrogate model is ViT-B/16.

Attacks	ViT-B/16	Swin-B	RVT	DenseNet-121	VGG-19	ResNet-50	Avg
MAN	20.7	6.1	1.6	2.4	2.5	2.0	5.9
CGNC	30.5	12.8	4.7	10.5	11.1	8.6	13.0
AMGAA	41.7	30.4	14.6	15.0	14.7	10.2	21.1

**Table 4:** Perceptual quality evaluation of adversarial examples.

Attacks	SSIM	LPIPS
G-Patch	0.85	0.024
CGNC	0.91	0.019
AMGAA	0.91	0.018

## Data Availability

The datasets used in this study are publicly available. CIFAR-10 is available at https://www.cs.toronto.edu/~kriz/cifar.html (accessed on 8 June 2026), and ImageNet is available at https://www.image-net.org/ (accessed on 8 June 2026). Further details and processed experimental results are available from the corresponding author upon reasonable request.
